# Quadriceps physiological response during the 1-min sit-to-stand test in people with severe COPD and healthy controls

**DOI:** 10.1038/s41598-022-04820-z

**Published:** 2022-01-17

**Authors:** Sarah Gephine, Patrick Mucci, Mathieu Bielmann, Mickael Martin, Laurent Bouyer, Didier Saey, François Maltais

**Affiliations:** 1 Institut Universitaire de Cardiologie et de Pneumologie de Québec, Université Laval, 2725 Chemin Sainte-Foy, Quebec, QC G1V 4G5 Canada; 2 Univ. Lille, Univ. Artois, Univ. Littoral Côte d’Opale, ULR 7369 - URePSSS - Unité de Recherche Pluridisciplinaire Sport Santé Société, Lille, F-59000 France; 3 Centre Interdisciplinaire de Recherche en Réadaptation et Intégration Sociale, Quebec, Canada

**Keywords:** Diseases, Circulation, Neurophysiology, Respiration

## Abstract

We compared quadriceps oxygenation and surface electromyography (sEMG) responses during the 1-min sit-to-stand (1STS) in 14 people with severe COPD and 12 controls, in whom cardiorespiratory response, near-infrared spectroscopy signals (oxy [Hb-Mb], deoxy [Hb-Mb], total [Hb-Mb], and SmO_2_) and sEMG signals of the quadriceps were recorded. Time duration of each sit-to-stand cycle and the total work performed during the 1STS were measured. The quadriceps oxygenation parameters were normalized by reporting their values according to the total work during 1STS. The rate of sit-to-stand maneuvers decelerated in people with COPD leading to smaller total work compared with controls. The pattern of quadriceps oxygenation response during 1STS was similar between groups. However, in COPD, the recovery after 1STS was characterized by larger overshoots in oxy [Hb-Mb], total [Hb-Mb], and SmO_2_. When corrected for the cumulative total work, the increase in muscle O_2_ extraction (deoxy [Hb-Mb]) during the first 30 s of recovery was greater in people with COPD compared to controls. Quadriceps sEMG changes suggestive of a fatiguing contraction pattern was observed only in people with COPD. All together, these results highlighted physiological misadaptation of people with severe COPD to the 1STS.

## Introduction

Reduced exercise capacity is a hallmark of chronic obstructive pulmonary disease (COPD). Several factors contribute to this situation, from the impairment in lung function that leads to constrain the capacity to expand tidal volume during exercise to limb muscle dysfunction that is commonly observed in COPD^1–3^. Limb muscle dysfunction, one of the most important systemic consequences of COPD, is characterized, amongst other things, by atrophy, changes in fiber-type distribution and oxidative metabolism^4,5^ that cumulate in reducing muscle strength and endurance, in addition to render the muscle susceptible to premature fatigue^3,6,7^.

Reflecting a common activity in daily living, the 1-min sit-to-stand test (1STS) is widely used in clinical practice as a valid and reliable field test which supposedly assesses the functional status of people with COPD^8,9^. Furthermore, its ability to predict two-year mortality risk enhances its clinical importance^10^. However, recent studies suggested that the 1STS elicits a rapid and near maximum physiological response associated with dynamic hyperinflation to which people with COPD have difficulties to adjust^8,11^. Indeed, we reported an oxygen uptake ($\dot{V}{\text{O}}_{2}$) overshoot and oxygen desaturation during the recovery phase of the 1STS in people with severe COPD^11^, illustrating that appropriate physiological adjustments to the 1STS do not occur during the active phase of the test.

Impaired quadriceps oxidative capacity could also contribute to the reduction in 1STS performance in people with COPD compared to healthy controls^11,12^. Previous studies indicated that, despite lower total muscle work, quadriceps deoxygenation during localized lower limb exercises is greater in people with COPD compared to healthy controls^13–15^. This observation suggests an impaired adjustment between quadriceps O_2_ delivery and utilization^12,15^. This is especially true for high intensity exercises, such as the 1STS, where dynamic hyperinflation, hypoxemia, pulmonary hemodynamics, and blood flow competition between the respiratory and limb muscles^16^ could slow down the limb oxygen delivery response time^17^. Along this line, the overshoot in $\dot{V}{\text{O}}_{2}$ that we reported during the recovery after 1STS^11^ could reflect an increase in peripheral muscle oxygen extraction to “repay” the oxygen deficit accumulated with exercise. This hypothesis was also supported by previous studies showing slower quadriceps deoxygenation and blood flow recovery kinetics in people with COPD compared to healthy controls after localized lower limbs exercise^18,19^. Thus, evaluating the quadriceps oxygenation changes during and after 1STS could help understanding the mechanisms of exercise intolerance in COPD.

We also reported that quadriceps contractile fatigue may occur during the 1STS in people with severe COPD^11^. Surface electromyography (sEMG) can also be used to track down the development of muscle fatigue during exercise^6,7,20^. For example, we previously reported that the fall in sEMG median frequency of the quadriceps could be used to indicate the occurrence of contractile fatigue of this muscle after cycling exercise in people with COPD^6^. However, this technique has never been used for measuring quadriceps fatigue during the 1STS. This may be clinically relevant since the profile of lower limb muscle activation during the 1STS may be used to discriminate healthy older with risk of falls^21^.

The present study was therefore undertaken to compare the quadriceps oxygenation and sEMG responses during the 1STS in people with COPD in comparison to healthy controls of similar age, sex, body mass index and physical activity status. We hypothesized that, compared to healthy controls, people with COPD would (i) reached higher quadriceps deoxygenation relative to the work performed during 1STS and, consistent with the previously reported $\dot{V}{\text{O}}_{2}$ overshoot during recovery, a higher quadriceps oxygen extraction during recovery after 1STS, and (ii) experience a higher fall in quadriceps sEMG median frequency, suggestive of a fatiguing contraction pattern during the 1STS.

## Methods

### Study design and participants

This was a cross-sectional, controlled study conducted at the *Institut universitaire de cardiologie et de pneumologie de Québec*, Québec, Canada. Fourteen people with severe to very severe COPD (forced expiratory volume in 1 s [FEV_1_] < 50% predicted values) and 12 healthy controls were recruited. People with COPD were eligible if they were at least 40 year of age, had a body mass index (BMI) < 30 kg·m^2^ , were former smokers with a cumulative tobacco exposure > 10 pack-years, and had a sedentary lifestyle defined by a Voorrips score < 9^22^. Exclusion criteria included any cardiovascular, neurological, neuromuscular, or orthopedic diseases that could affect the ability to perform study procedures; current asthma; participation in pulmonary rehabilitation in the past 6 months; and being on > 10 mg daily prednisone equivalent. Apart from lung function criteria, the same inclusion and exclusion criteria were applied to people with COPD and healthy controls to have two groups paired for sex, age, BMI, and level of physical activity. The protocol and the characteristics of the same study participants have already been published elsewhere^11^, but apart from peak cardiorespiratory response data which are necessary for the understanding of this study, data presented in this manuscript are original.

### Procedure

The study consisted in one visit during which anthropometric measurements were assessed by bioelectrical impedance (InBody520, Body Composition Analyzer, Seoul, Korea) and pulmonary function tests, including spirometry, plethysmographic lung volumes, and carbon monoxide diffusion capacity were performed according to standard guidelines^23^. The 1STS was performed after 30 min of rest following the pulmonary function tests as previously described^11^. Participants were asked to sit with the knees and hips flexed to 90°, feet placed flat on the floor, and hands placed on the hips. Standardized instructions informed study participants to stand completely straight and immediately sitting back as many time as possible in one minute without using hands. Performance of 1STS was reported by the number of repetitions and the associated total work was estimated (number of repetitions*body mass [kg]).

### Measurements

#### Cardiorespiratory monitoring

At rest, during the 1STS, and during four minutes of recovery, oxygen uptake ($\dot{V}{\text{O}}_{2}$) was measured breath-by-breath, and heart rate (HR) and pulse oxygen saturation (SpO_2_) were monitored beat by beat with a portable gas analysis system (Oxycon Mobile; Viasys Healthcare, Jaeger, Germany), as previously described^11^. The analyzer was calibrated before each test according to the manufacturing recommendations.

#### Quadriceps oxygenation

A continuous wave multichannel near-infrared spectroscopy (NIRS) system (OxiplexTS, ISS, Champaign, USA) was used at two wavelengths in the near-infrared range to detect quadriceps changes in absolute concentration of oxygenated (Δ oxy [Hb-Mb]), deoxygenated (Δ deoxy [Hb-Mb]), total (Δ total [Hb-Mb]) myoglobin–hemoglobin concentration, and muscle saturation index (Δ SmO_2_) during the 1STS, and during 4 min of recovery. The NIRS fiber optode consisted of eight light-emitting diodes operating at wavelengths of 690 and 830 nm with interoptode distances of 2.5, 3.0, 3.5, and 4.0 cm. Based on a 50% ratio between interoptode distance and penetration depth^24^, the experimental setup allowed for a penetration depth of 1.25 to 2 cm. The NIRS probe calibration was verified prior to each testing session according to the manufacturer’s recommendations, using a calibration block of known absorption and scattering coefficients. To avoid the influence of room light, probe was covered with an optically black band^24^ and fixed on the fleshy part of the quadriceps, below the EMG electrode. Prior to testing, the adipose tissue thickness of the leg was measured using skin calipers (Baseline Skinfold Caliper, NexGen Ergonomics, Canada).

#### Electromyography

Surface electromyography signal (sEMG) from the right quadriceps was recorded throughout the 1STS (FreeEMG300, BTS Bioengineering, Milan, Italy) with a bioelectric signal amplifier, wireless transmission, and bipolar electrodes. The sEMG signal was high-pass-filtered (1 kHz) and preamplified near the recording electrodes. Electrodes were placed on the muscle bellies, longitudinally with respect to the underlying muscle fibers arrangement and were located according to the surface electromyography for the non-invasive assessment of muscles (SENIAM) recommendations^25^. Before placing the electrodes, the electrical impedance of the skin was reduced by shaving the hair and by cleaning the skin with alcohol. An electrogoniometer (FreeEMG300, BTS Bioengineering, Milan, Italy) was placed on the participant’s left leg to record and dissociate the sitting and standing phases during the 1STS.

### Data and statistical analyses

The number of repetitions and the duration of each sit-to-stand were counted using the electrogoniometer signal. The mean duration of two sit-to-stand at 15 s, 30 s, 45, and 60 s of 1STS was calculated. The cumulative total work at 15 s, 30 s, 45, and 60 s of 1STS was calculated by multiplying the cumulative number of repetitions performed at each time by the individual’s body mass.

Breath-by-breath cardiorespiratory data recording was synchronized to muscle oxygenation at 1-s intervals. Therefore, simultaneously recorded data were obtained at rest, and during the 1-min exercise, and four minutes of recovery. Because of the short duration of the 1STS, end-exercise cardiorespiratory parameters are reported from the mean of the last two breaths.

Changes in Δ oxy [Hb-Mb], Δ deoxy [Hb-Mb], Δ total [Hb-Mb], and Δ SmO_2_ were calculated with the respective baseline value as reference. On the basis of the Fick principle, Δ deoxy [Hb-Mb] responses reflects the dynamic balance between oxygen delivery and consumption in the investigated muscle, providing an index of fraction oxygen extraction in local muscle, whereas Δ total [Hb-Mb] can be interpreted as an indirect estimation of local blood volume in the tissue^26^. These data were imported into a personal computer at a sampling frequency of 1 Hz via an analog-to-digital converter (PowerLab, ADInstruments, Australia) allowing synchronization with breath-by-breath cardiorespiratory data. Muscle oxygenation responses were expressed as percent change from resting values. Δ oxy [Hb-Mb], Δ deoxy [Hb-Mb], Δ total [Hb-Mb], and Δ SmO_2_ were averaged over the last 15 s of rest before the test (baseline data) and the last 15 s of each 30-s period during the recovery (90, 120, 150, 180, 210, 240, 270, 300 s). Because of the 1STS short duration, these data were also averaged over the last 3 s at 15, 30, 45, and 60 s of 1STS. To take into account difference in the 1STS total work between people with COPD and healthy controls, Δ oxy [Hb-Mb], Δ deoxy [Hb-Mb], Δ total [Hb-Mb], and Δ SmO_2_ were also expressed by dividing their value by the cumulative work during 1STS and during four minutes of recovery. Results are expressed as mean ± SEM.

All sEMG signals were analysed using custom software written in MATLAB R2018a (The Math Works Inc., Natick, Massachusetts, United States). The signals were digitally filtered off-line with a zero lag fourth order Butterworth filter (band-pass 20–450 Hz) and amplitude analysis was performed using a root mean square-based envelope (root mean square of a non-overlapping 20 ms rectangular window). The signals were cut and time-normalized for each sit-to-stand (one cycle) using the electrogoniometer signals during 1STS. Mean of sEMG envelopes was calculated for each cycle. Frequency analysis was performed on digitally filtered data prior to root mean square envelope and time normalization steps. Power spectral median frequency was calculated for each cycle and time duration of each cycle was extracted.

Sample size calculation was based on Ribeiro et al.^13^, to detect a quadriceps Δ deoxy [Hb-Mb] difference between people with COPD and healthy controls of 15 ± 18% at the end of 1STS, with a power of 80% and an alpha of 5%. With this method, the sample size was calculated to be 14 participants in each group. Descriptive data was expressed as mean ± standard deviation, and statistical significance was considered at *p* < 0.05. All variables were tested for normality using Shapiro-Wilks test. Non-normally distributed data were log-transformed before analysis. Paired t-tests were used to evaluate between-group differences in baseline characteristics, 1STS performance, peak cardiorespiratory responses and symptom perception during 1STS. Two-way repeated measure ANOVA, with Holm-Bonferroni post hoc corrections, were used for within-group quadriceps oxygenation and sEMG data across time points. A mixed model analysis was performed with an interaction term between groups and time. SigmaPlot 11.0 (Systat Software, San Jose, California) was used for statistical analyses.

### Ethics approval and consent to participate

The study was conducted in accordance with the declaration of Helsinki and was approved by the *Comité d’éthique de l’Institut universitaire de cardiologie et de pneumologie de Québec, Université Laval* (CER: 21539). All participants received written and verbal information about the study and gave their written informed consent before the study commenced.

## Results

**Figure 1:**
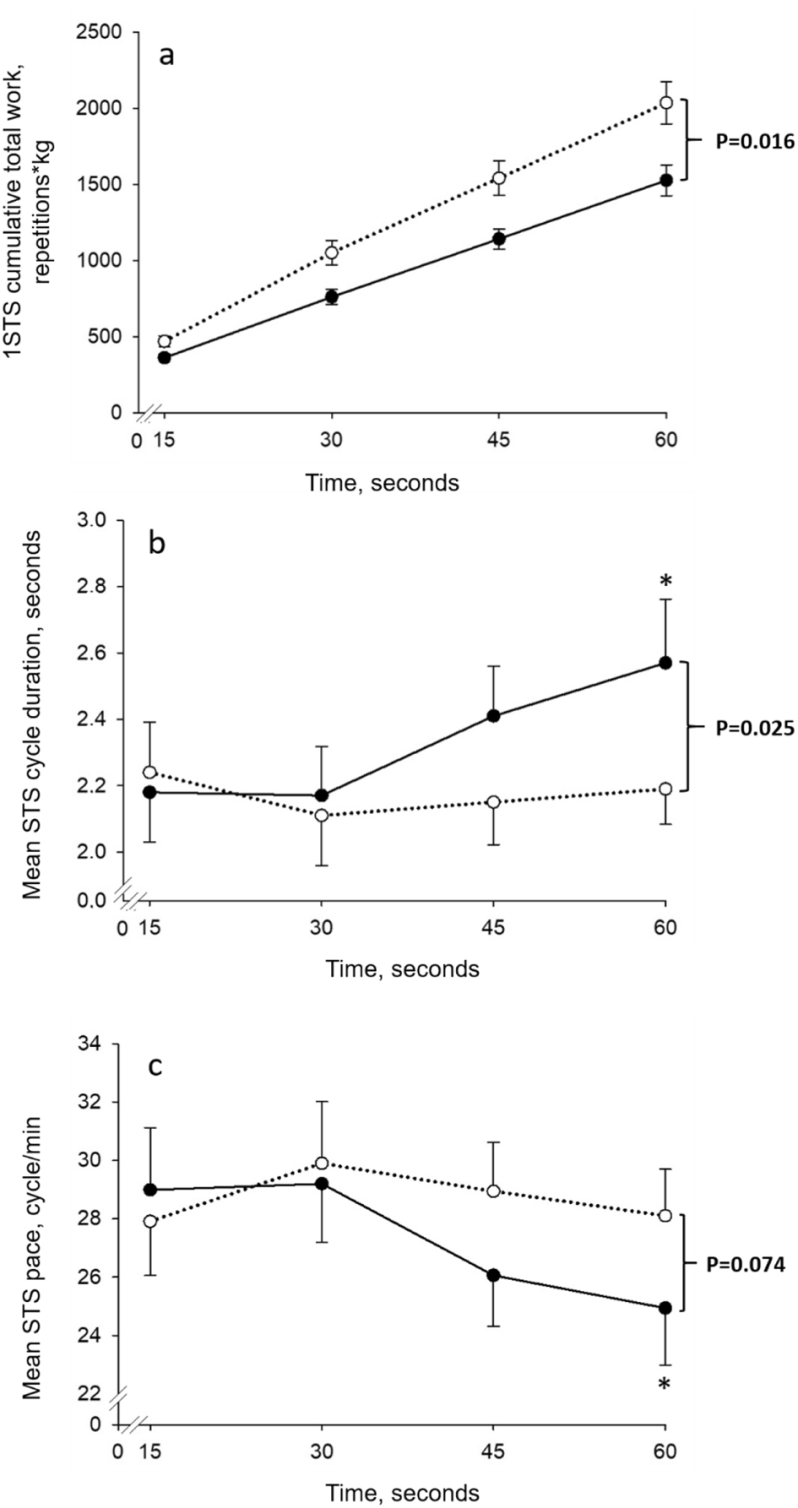
Time course of cumulative total work during the 1-min sit-to-stand (1STS) (**a**), mean duration time of two sit-to-stand cycles at 15 s, 30 s, 45 s, and 60 s of the 1STS (**b**), and mean pace during the 1STS at 15 s, 30 s, 45 s, and 60 s of the 1STS (**c**) in people with COPD (full circles) and healthy controls (open circles). Results are presented as mean ± SD. Data were not recorded for three people with COPD and four healthy controls because of technical problems with the electrogoniometer. Overall, the pattern of changes in cumulative total work differred between groups during 1STS (*p* = 0.016) (**a**) as a result of a progressive deceleration during 1STS that was observed only in people with COPD (*p* < 0.01). *p < 0.01, 60 s compared to 15 s. The *p* values report the interaction group × time during the 1STS.

Characteristics and 1STS performance data of study participants are presented in Table 1. As expected from the experimental design, age, sex, BMI, and level of physical activity were similar between groups. People with COPD had lower pulmonary function, 1STS number of repetitions (24 ± 5 vs 31 ± 6, *p* < 0.01) and cumulative total work during the 1STS than healthy controls (Fig. 1a). The mean duration time and mean pace of two sit-to-stand at 15, 30, 45 and 60 s of the 1STS in people with COPD and healthy controls are presented in Fig. 1b,c, respectively. A progressive deceleration during the 1STS was observed only in people with COPD; in these individuals, an average sit-to-stand cycle took 0.40 s longer in the last 15 s of the 1STS compared to the first 15 s (*p* < 0.01), resulting in a slower pace during the 1STS.

**Table 1:** Characteristics of study participants.

	COPD (n = 14)	Healthy controls (n = 12)	*p* value
Age, years	65 ± 8	65 ± 7	0.633
Female, n (%)	7 (50)	5 (42)	0.347
BMI, kg/m^2^	25 ± 3	24 ± 2	0.723
FFM, %	39 ± 5	37 ± 4	0.076
**Smoking status, n (%)**			< 0.001
Former	14 (100)	3 (25)	
Never	0 (0)	9 (75)	
FEV_1_, % of predicted	38 ± 8	119 ± 13	< 0.001
FVC, % of predicted	88 ± 12	127 ± 15	< 0.001
FEV_1_/FVC, %	35 ± 7	76 ± 4	< 0.001
RV, % predicted	159 ± 40	78 ± 14	< 0.001
DLCO, % predicted	45 ± 11	80 ± 13	< 0.001
mMRC dyspnea scale (0–4)	2.7 ± 0.5	1.7 ± 0.5	< 0.001
Voorrips physical activity	5.6 ± 2.0	7.3 ± 2.8	0.172
1STS, number of repetitions	24 ± 5	31 ± 6	0.009
1STS, total work, repetitions*kg	1603 ± 517	2011 ± 540	0.006

Data are presented as mean ± SD or number (percentages).

*FFM* Fat-free mass, *FEV*_*1*_ Forced expiratory volume in 1 s, *FVC* Forced vital capacity, *RV* Residual volume, *DLCO* Diffusion capacity of the lung for carbon monoxide, *mMRC* Modified Medical Research Council, *1STS* 1-min sit-to-stand test.

### Cardiorespiratory response

Peak cardiorespiratory parameters were greater in healthy controls compared to people with COPD ($\dot{V}{\text{O}}_{2}$, 18.7 ± 3.6 vs 14.2 ± 2.0 ml kg^−1^ min^−1^; heart rate 126 ± 14 vs 110 ± 13, *p* < 0.01). In people with COPD, peak $\dot{V}{\text{O}}_{2}$ was reached during recovery and occurred 16 ± 13 s after the end of exercise. Such an overshoot was not observed in healthy controls in whom peak $\dot{V}{\text{O}}_{2}$ was reached at the end of 1STS. During 1STS, a ≥ 4% SpO_2_ fall was seen in seven people with COPD, amongst whom nadir SpO_2_ value was reached during recovery period in five individuals (mean rest SpO_2_ value: 95 ± 3%; mean fall in SpO_2_: -5 ± 4%). None of the healthy controls showed a ≥ 4% SpO_2_ fall (mean rest SpO_2_ value: 96 ± 2%; mean fall in SpO_2_: -1 ± 2%). Additional details about the cardiorespiratory response during 1STS can be found elsewhere^11^.

### Quadriceps oxygenation during 1STS

**Figure 2:**
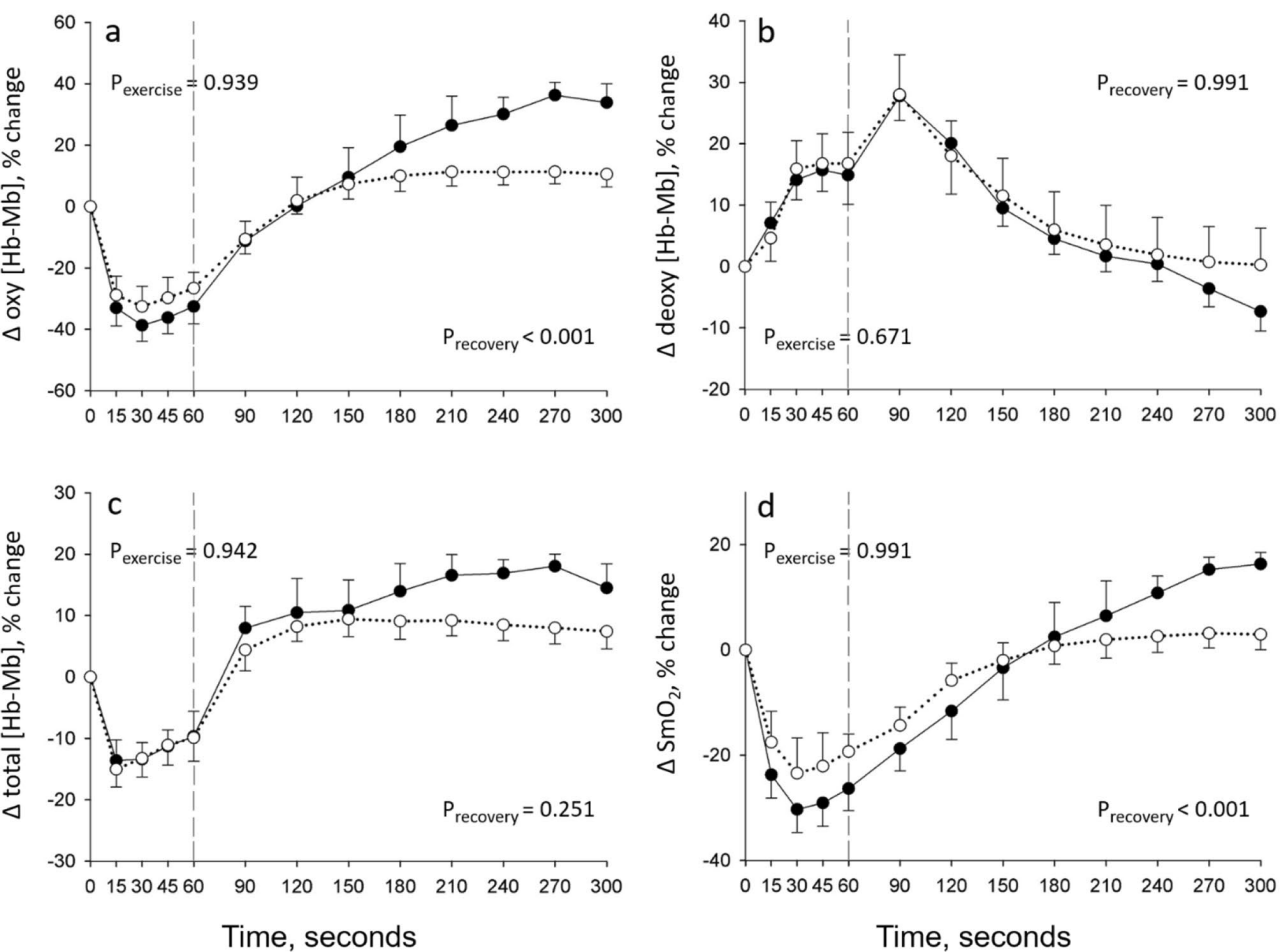
Time course of quadriceps (**a**) oxygenated (Δ oxy [Hb-Mb]), (**b**) deoxygenated (Δ deoxy [Hb-Mb]), (**c**) total (Δ total [Hb-Mb]) myoglobin–hemoglobin concentration, and (**d**) muscle saturation index (Δ SmO_2_) during the 1STS and 4 min of recovery in people with COPD (full circles) and healthy subjects (open circles). Results are presented as mean ± SEM. Exercise and recovery data were analysed with two distinct two-way repeated measure ANOVAs, the *p* values report the interaction group × time during the 1STS and during the recovery periods, separately.

**Figure 3:**
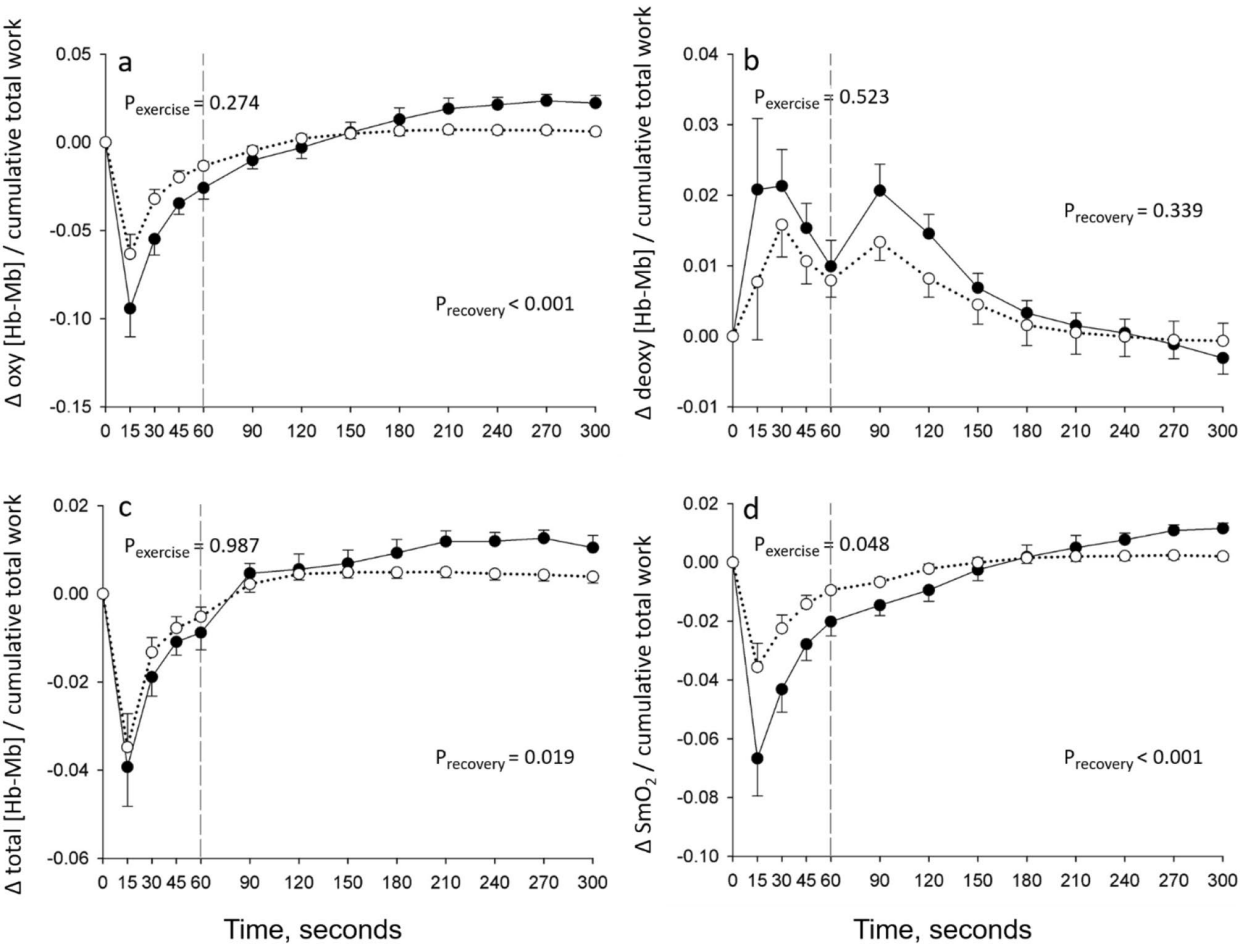
Time course of quadriceps (**a**) oxygenated (Δ oxy [Hb-Mb]), (**b**) deoxygenated (Δ deoxy [Hb-Mb]), (**c**) total (Δ total [Hb-Mb]) myoglobin–hemoglobin concentration, and (**d**) muscle saturation index (Δ SmO_2_) per unit of cumulative total work during the 1STS and 4 min of recovery in people with COPD (full circles) and healthy subjects (open circles). Results are presented as mean ± SEM. Exercise and recovery data were analysed with two distinct two-way repeated measure ANOVAs, the *p* values report the interaction group x time during the 1STS and during the recovery periods, separately.

Quadriceps oxygenation was not measured in one patient with COPD and one healthy control because of technical problems. The adipose tissue thickness over the quadriceps muscle belly was 4.7 ± 2.1 mm 5.3 ± 1.9 mm for patients with COPD and healthy controls, respectively. Compared to baseline, there was a rapid fall in Δ oxy [Hb-Mb], Δ total [Hb-Mb], and Δ SmO_2_ in both groups during the 1STS (*p* < 0.001); these parameters tended to plateau for the remaining of the test (Fig. 2a,c,d). These changes were of similar magnitude between people with COPD and healthy controls. Δ deoxy [Hb-Mb] rapidly increased in both groups during the first 30 s of the 1STS (*p* < 0.05) with a subsequent plateau for the remaining of the 1STS. The amplitude of the changes in Δ deoxy [Hb-Mb] during 1STS was similar between groups (Fig. 2b). As illustrated in Fig. 3, expressing the quadriceps oxygenation data taking into account cumulative work during the 1STS did not alter the conclusions, with the exception of Δ SmO_2_ whose decrease per unit of cumulative work during the 1STS was more pronounced in people with COPD compared with healthy controls (*p* = 0.048).

### Quadriceps oxygenation during recovery

After falling during the 1STS, Δ oxy [Hb-Mb], Δ total [Hb-Mb], and Δ SmO_2_ increased during the recovery period of the 1STS (*p* < 0.05), a pattern that was similarly seen in both groups (Fig. 2a,c,d). At variance, Δ deoxy [Hb-Mb] continue to increase during the first 30 s of recovery after 1STS (*p* < 0.001, compared to the end of 1STS), following by a progressive return to the resting values after 90 s of recovery (Fig. 2b). The overshoot in Δ oxy [Hb-Mb] and Δ SmO_2_ responses was more pronounced in people with COPD compared with healthy controls (*p* < 0.001), while the Δ deoxy [Hb-Mb] and Δ total [Hb-Mb] responses were not different between groups (*p* = 0.991 and *p* = 0.251, respectively) (Fig. 2). Expressing the quadriceps oxygenation data taking into account cumulative work during the 1STS did not alter the results, with the exception of Δ deoxy [Hb-Mb] whose increase per unit of cumulative work during the first 30 s of recovery after 1STS was more pronounced in people with COPD compared with healthy controls (*p* = 0.009) (Fig. 3b).

**Figure 4:**
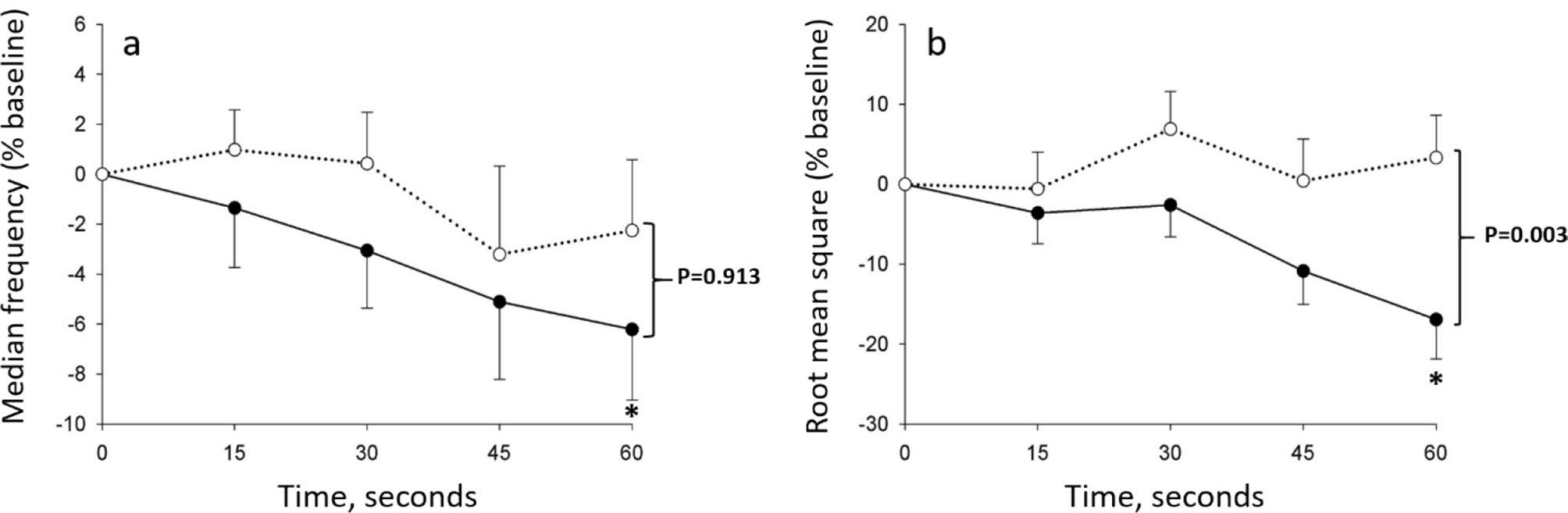
Changes in quadriceps (**a**) median frequency and (**b**) root mean square during the 1-min sit-to-stand (1STS), expressed in % baseline value in people with COPD (full circles) and healthy controls (open circles). Results are presented as mean ± SEM. *p < 0.01, 60 s compared to 15 s. The *p* values report the interaction group × time during the 1STS.

### Electromyography

Quadriceps sEMG was not recorded for three people with COPD and four healthy controls because of technical problems. In people with COPD, there was a fall in quadriceps median frequency during 1STS (6.9% ± 2.4%, *p* < 0.01) (Fig. 4), while in healthy controls the median frequency of the muscle remained stable during the test. Despite these within-group differences, the overall pattern of changes in median frequency did not differ between COPD and healthy controls (*p* = 0.913) (Fig. 4a). Root mean square of the quadriceps decreased during 1STS in people with COPD (*p* < 0.01), while it remained stable in healthy controls (Fig. 4b). The overall pattern of changes in root mean square was statistically different between people with COPD and healthy controls (*p* = 0.003) (Fig. 4b).

## Discussion

The novel findings of this study are as follows: (i) during the 1STS, people with COPD developed a progressive deceleration in the rate of sit-to-stand maneuvers that led to smaller cumulative total work during the 1STS compared with healthy controls; (ii) the quadriceps oxygenation response during 1STS was characterized by evidence of decreased muscle oxygenation (reduced oxy [Hb-Mb]) and increased muscle O_2_ extraction (increased deoxy [Hb-Mb]); this pattern was similar between people with COPD and healthy controls, with the exception of a more profound muscle desaturation when corrected for the cumulative total work in the former individuals; (iii) larger overshoots in quadriceps oxygenation and saturation were seen in people with COPD compared to healthy controls during the recovery after 1STS. When corrected for the cumulative total work, the increase in quadriceps O_2_ extraction during the first 30 s of recovery was higher in people with COPD compared to healthy controls; (iv) there was a diminution in quadriceps median frequency and root mean square during the second half of the 1STS only in people with COPD, suggestive of a fatiguing contraction pattern and a progressive reduction in quadriceps activation during the 1STS. Cumulatively, the present and previous findings^11^ highlight physiological misadaptation in people with severe COPD to this stressful exercise.

During the 1STS, quadriceps oxygenation changes were similar to what is seen with other exercise modalities, showing reduced oxy [Hb-Mb] and increased deoxy [Hb-Mb]^13–15^. The changes in Δ oxy [Hb-Mb], Δ deoxy [Hb-Mb], total [Hb-Mb], and Δ SmO_2_ were similar in people with COPD and healthy controls, supporting previous results^14,15^. Since cumulative total work during 1STS was lower in people with COPD compared to healthy controls, it seemed adequate to normalize the quadriceps oxygenation responses by this parameter. Although we found a similar pattern of response when cumulative work was considered, the diminution in quadriceps Δ SmO_2_ per unit of cumulative total work was higher in people with COPD compared with healthy controls. The quadriceps oxygenation findings during the 1STS were at variance with previous studies that have used other exercise modalities to report greater quadriceps deoxygenation per work performed, during lower limbs exercises in people with COPD compared to controls^13–15^. There are several potential explanations for these discrepant findings about quadriceps deoxygenation between the 1STS and other exercise modalities involving the lower limbs. First, although quadriceps are mainly involved during the sit-to-stand procedure, others muscles such as the tibialis anterior or rectus femoris also contribute to the performance^27^, while with other exercise modalities such as electrical stimulation or leg extension exercise, the quadriceps is the main muscle involved^13–15^. It should be highlighted that the general trend for a larger muscle deoxygenation during the 1STS in people with COPD was in the same direction as seen with other exercise modalities, but several reasons could have reduced the magnitude of the differences between the two groups. We previously reported evidences of cardiorespiratory system limitations during the 1STS in people with COPD^11^ that could explain the progressive reduction in the sit-to-stand cadence and in the metabolic demand of the quadriceps. The short duration of the 1STS could also have contributed to minimize the between-group difference in quadriceps oxygenation status. Lastly, the relatively small sample might have compromised by our ability to discriminate the two groups for small between-group differences in muscle oxygenation.

We previously reported, a $\dot{V}{\text{O}}_{2}$ overshoot and pulse oxygen desaturation during the first 30 s of recovery after 1STS in this group of people with COPD^11^. These results were interpreted as indicative of the inability of the cardiovascular and respiratory systems to adapt to a brutal increase in metabolic demand during the 1STS and to the necessity of ‘repaying’ the O_2_ deficit accumulated during the active phase of the 1STS during the recovery period^11^. Consistent with this, we report herein a rebound in Δ deoxy [Hb-Mb] per unit of cumulative total work during the first 30 s of recovery after 1STS in people with COPD. This phenomenon may be related to an altered peripheral O_2_ supply during the active phase of the 1STS, as previously reported in COPD^15,17,28^. The increase in total [Hb-Mb] during recovery was higher in people with COPD compared with healthy controls, suggestive of a pronounced post-exercise hyperemia, possibly related to the reduced SpO_2_ considering that mild hypoxia is a potent vasodilator^29^.

People with COPD experienced an absolute quadriceps median frequency fall of 7%, suggestive of a fatiguing contraction pattern. Indeed, a > 4% median frequency fall during a constant work-rate cycle exercise was established as a good clinical predictor of quadriceps contractile fatigue in COPD^6^. Consistent with this, we previously reported in the same individuals that two-thirds of them developed quadriceps contractile fatigue, assessed by magnetic stimulation, after the 1STS^11^. The root mean square of sEMG signals reflects the number of motor units recruited during an effort^20^. At variance to constant work-rate cycling exercise or local quadriceps contractions in people with COPD^6,30^, we found a progressive reduction in the root mean square of the vastus lateralis during the 1STS. The likely explanation for this discrepancy between constant work-rate exercises and the 1STS resides in the fact that the working intensity was not constant during 1STS as evidenced by the progressive deceleration observed only in people with COPD. In this context, the decrease in quadriceps root mean square during the last 30 s of 1STS in people with COPD would rather reflect the diminution in the intensity of the muscle contraction than the number of motor units recruited.

### Methodological considerations

The present results are not necessarily generalizable to patients with milder forms of COPD. Although deoxy [Hb-Mb] is considered as a good index of limb O_2_ extraction^28,31^, it may be influenced by muscle blood flow and systemic oxygen delivery that were not directly measured. We acknowledge that several physiological variables that were not controlled or measured during the experimental protocol may have influenced the fall in muscle oxygenation, although their relative contribution to the between-group difference is of uncertain significance and difficult to quantify. Muscle oxygenation is modulated by the rate of oxygen delivery and oxygen extraction^32^ and it could be argued that impaired cardiac output or blood flow to the exercising muscles during the 1STS in people with COPD may have contributed to the present results. People with COPD with no known cardiovascular comorbidity were selected for this study but we cannot exclude that undiagnosed cardiovascular disease was present in some of them^33^. To the same extend, a greater rate of lactate production and muscle acidosis during the 1STS in people with COPD compared to healthy controls may have influenced the changes in quadriceps oxygenation though the Bohr effect, although the lower amount of work performed in the former group should have mitigated this effect. Lastly, between-group differences in the rate of muscle contraction which was not controlled in this study, could have confounded the muscle O_2_ saturation measurements. Finally, cautious is warranted when interpreting the statistical significances of the sEMG data because the sample size was not calculated according to this parameter.

## Conclusion

During 1STS, deceleration in the rate of sit-to-stand maneuvers was seen in people with COPD, leading to smaller cumulative total work compared with healthy controls. While we previously reported evidences of cardiopulmonary limitations in this population during the 1STS^11^, the quadriceps oxygenation and sEMG data nevertheless suggest that the 1STS was also challenging at the muscle level. This was further exemplified during recovery after the 1STS which was characterized by an increased in quadriceps O_2_ extraction concomitantly to an $\dot{V}{\text{O}}_{2}$ overshoot and a hyperemia observed in people with COPD.

## Data Availability

The datasets used and/or analyzed during the current study are available from the corresponding author on reasonable request.
